# Exposure to antenatal corticosteroids and infant cortisol regulation

**DOI:** 10.1016/j.psyneuen.2022.105960

**Published:** 2022-10-26

**Authors:** Sandra J. Weiss, Victoria Keeton, Sarah Richoux, Bruce Cooper, Sandra Niemann

**Affiliations:** a Department of Community Health Systems, University of California, San Francisco, USA; b Department of Obstetrics, Gynecology & Reproductive Sciences, University of California, San Francisco, USA

**Keywords:** Corticosteroids, HPA axis, Cortisol, Fetal programming, Pregnancy Infant

## Abstract

Administration of antenatal corticosteroids (AC) is the standard of care during pregnancy for women who are at risk of early delivery. Evidence indicates that AC improve survival and reduce morbidity for preterm infants. However, research suggests that infants whose mothers receive AC have an altered hypothalamic-pituitary-axis (HPA) response to stressors in early life. Results are mixed regarding the nature of these effects, with studies showing both suppressed and augmented HPA activity. In addition, research is very limited beyond the 4th month of life. The purpose of this study was to determine if AC exposure was associated with infant cortisol levels in a resting state or in response to a stressor at 1, 6 and 12 months postnatal. We also evaluated the moderating role of preterm birth in this association. 181 women and their infants participated in the study. Women were recruited during the 3rd trimester of pregnancy; at this time, they completed the Perceived Stress Scale and provided 8 salivary samples over a 2-day period for cortisol assay. They provided these data again at 6 and 12 months postnatal. At 1, 6, and 12 months postnatal, salivary samples were collected from infants to examine their cortisol levels before and after participation in a ‘stressor protocol’. Data were extracted from the medical record on AC exposure, gestational age, maternal obstetric risk, and neonatal morbidity. Mixed effects multilevel regression modeling was used to examine the aims. Infants whose mothers received AC had significantly lower resting state (B = −2.47, CI: −3.691, −0.0484) and post-stressor (B = −.51, CI: −4.283, −0.4276) cortisol levels across the first year of life than infants whose mothers did not receive AC. There was no moderating effect of preterm birth on the relationship between AC exposure and cortisol. Results indicate a state of dampened HPA activation and cortisol hypo-arousal that persists across the first year of life among infants who were exposed to corticosteroids in utero. Further research is needed to examine mechanisms responsible for any alterations that occur during development of the fetal HPA axis, including epigenetic and biochemical factors that control hormonal secretion, negative feedback, and glucocorticoid receptor function throughout the HPA axis. Findings warrant careful consideration by obstetric clinicians of the benefits and risks of prescribing AC.

## 1. Introduction

### 1.1. Antenatal corticosteroids: benefits and risks

Since 1994, antenatal corticosteroids (AC) have been the standard of care for women who are fewer than 34 weeks gestation and experiencing threatened preterm birth. Guidelines have expanded recently to include women at later gestation with select risk factors (Society for Maternal-Fetal Medicine (SMFM), 2016). These synthetic glucocorticoids (betamethasone or dexamethasone) are given to mimic the surge in endogenous corticosteroids that is essential for development of many organ systems and prepares the fetus for extra-uterine life (Kemp et al., 2018). Substantial evidence demonstrates that a single course of AC improves survival and reduces morbidity for preterm infants (McGoldrick et al., 2020; Roberts et al., 2017). But research in animals and humans has raised concerns about potentially harmful long-term effects (Ilg et al., 2019; Tegethoff et al., 2009; Waffarn and Davis, 2012). Because AC expose the fetal brain to high levels of glucocorticoids (GC) early in development, they may exert programming effects on the hypothalamic-pituitary-adrenal (HPA) axis (McGowan and Matthews, 2018; Moisiadis and Matthews, 2014), influencing an infant’s general state of arousal or ability to regulate emotional distress (Davis et al., 2011; Niwa et al., 2020; Schäffer et al., 2009; Weiss and Niemann, 2015). Such programming could permanently alter an infant’s resilience or vulnerability to stress and thus compromise long-term health. These effects could be more significant for premature infants, whose neurodevelopment and biological readiness for stressors are immature at birth (Lammertink et al., 2020).

### 1.2. Effects of AC on cortisol and stress regulation

Human research examining the effects of AC on the HPA axis has focused primarily on infancy, although evidence is beginning to accumulate about longer-term effects in school-age children. Evidence in the early postnatal period has been somewhat mixed, with the majority of studies demonstrating that AC exposure is associated with a suppressed HPA axis response in infants shortly after birth (Buyukkayhan et al., 2009; Cabral et al., 2013; Davis et al., 2004, 2006; Hwang et al., 2019; Karlsson et al., 2000; Schäffer et al., 2009; Weiss and Niemann, 2015). However, others have found infants to exhibit elevated basal cortisol levels (Davis et al., 2011; Kajantie et al., 2004; Nykänen et al., 2007) or no significant associations at all (Ng et al., 2019; Teramo et al., 1980). Few studies have examined the influence of gestational age (GA) in effects of AC on cortisol, and those have only investigated samples of preterm infants. One study of very preterm infants (born at < 28 weeks gestation) found that GA, but not AC, was significantly associated with basal cortisol levels (Ng et al., 2019), while two others reported no moderating effects of AC on basal cortisol (Ashwood et al., 2006; Karlsson et al., 2000).

Studies of older infants and school-age children demonstrate that suppression of basal cortisol levels does not appear to be present beyond the postnatal period. Instead, those exposed to AC show either elevated (Erni et al., 2012; Glover et al., 2005; Niwa et al., 2020) or similar (Alexander et al., 2012; Gover et al., 2012; Ilg et al., 2019; Miller et al., 2004) basal cortisol levels when compared to children not exposed. The only study examining cortisol in adults who had been exposed to AC found no significant associations (Dalziel et al., 2005).

Evidence is more limited for associations between AC exposure and stress reactivity. Infants exposed to AC exhibit alterations to stress reactivity in response to immunization, demonstrated by either suppression (Glover et al., 2005) or upregulation (Niwa et al., 2020) of the HPA axis. One study explored the moderating effect of gestational age on the relationship between AC and cortisol reactivity in a group of preterm vs. late preterm infants (Ashwood et al., 2006). Investigators found significantly greater suppression of cortisol in response to a stressor for infants born younger than 32 weeks gestation in contrast to late preterm infants, but there was no comparison to infants who had not received AC (Ashwood et al., 2006). Results of research beyond infancy indicate that school-age children exposed to AC have greater stress reactivity compared to those not exposed (Alexander et al., 2012; Erni et al., 2012), a finding that persists into adolescence (Ilg et al., 2019).

Taken together, most studies show an altered cortisol response in children exposed to AC, evident in the first days of life as HPA suppression and then re-emerging in school age and adolescence as increased stress reactivity. However, differential effects of AC on basal levels of cortisol versus effects on cortisol reactivity to stressors remain unclear. In addition, progression of potential cortisol dysregulation over the first year of life is understudied. Further research is needed to ascertain how long suppression may persist and explore the trajectory of HPA axis alteration from birth to one year of life. Virtually no studies have included samples of both term and preterm infants large enough to examine differential effects of AC on cortisol levels or reactivity of these two groups. Examining the potential moderating role of prematurity in any effects of AC is critical to better understand how prematurity and AC may interact in eliciting alterations in the HPA axis and to whom any effects of AC can be generalized.

Our study had two aims. Our primary aim was to determine if AC exposure is associated with infant resting state cortisol, post-stressor cortisol, or cortisol reactivity to a stressor at 1 month, 6 months, or 12 months postnatal. Our hypothesis was that infants exposed to corticosteroids would have significantly dampened cortisol at rest and in response to a stressor across all time periods, when compared with infants who were not exposed to AC during gestation. Our secondary aim was to determine if preterm birth status moderates the relationships between AC exposure and infant cortisol in a resting state or in reactivity to a stressor. We hypothesized that preterm infants would experience greater dampening of their cortisol levels and cortisol reactivity than term infants from exposure to AC.

## 2. Materials and methods

### 2.1. Study design and recruitment

This research was part of a larger longitudinal study that examined the effect of various risk factors during pregnancy on birth outcomes and the stress regulation of infants over the first year of life. We performed a power analysis to determine needed sample size. Based on findings from our previous research and that of others, we estimated that the difference between AC exposed and not exposed infants would be a medium effect at baseline (d =0.5) and between a medium and large effect over time (d =0.64). The interaction between AC group and preterm status was estimated as a medium effect at baseline (d =0.5), and between a weak and medium effect over time (d =0.35). Power estimations were obtained with Monte Carlo simulations and carried out with 10,000 random draws. We determined that effects specified in our aims could be detected by enrolling a sample size of 136, with power at or above .80 and two-sided alphas at .05. Our actual sample size for this analysis was n = 181 infants.

Mothers who were 18 years of age or older were recruited from two Obstetrics and Gynecology clinics affiliated with a large university medical center. Inclusion criteria were that women spoke English or Spanish, were in their third trimester of pregnancy (between 24 and 34 weeks’ gestation) and were identified by their obstetric clinician as being at increased risk for preterm labor. Risk for preterm labor was based on factors such as history of preterm birth and current obstetric health status. However, women who delivered at term were retained in the study along with those who delivered prematurely. Women who had ongoing steroid use or a history of endocrine conditions, those who smoked, and women with serious medical problems (e.g. cancer, cardiomyopathy) or cognitive impairment were excluded. Additionally, infants with chromosomal and genetic anomalies, chronic lung disease, congenital heart disease, or other major neonatal illness were excluded. Data of children being treated with synthetic glucocorticoids after birth (e.g. for asthma) or who developed endocrine problems (e.g. congenital adrenal hyperplasia) were not included in the analysis. A member of the research team contacted eligible women to provide details about the study and obtain informed consent if they were interested. Proxy consent for their infants to participate was obtained from the pregnant women. The study was approved by the University Institutional Review Board for Human Research Protection.

During the week of recruitment, women completed a baseline sociodemographic questionnaire and the Perceived Stress Scale (PSS). They also received written, verbal, and behavioral guidance on how to provide saliva samples into a small vial. They provided saliva samples 4 times each day over a 2-day period during pregnancy, and again for 2 days at 6 and 12 months postpartum. After the infant’s birth, we collected data from women and their infants during home visits at 3 timepoints: 1, 6, and 12 months of infant age. At each of these visits, a ‘stressor’ protocol was implemented (described below) to measure infant cortisol levels before and after the stressor. The researcher prepared mothers for the stressor ahead of the visit and discussed exactly what the protocol would involve. Mothers identified a suitable, quiet time and area in the home where the protocol could take place without disturbance by others living at the residence.

Data were extracted from the electronic medical records of mothers and infants to identify information about covariates for potential inclusion in testing of the study aims. Information was acquired regarding receipt of antenatal corticosteroids as part of obstetric care, overall maternal obstetric risk, infant gestational age, preterm birth status, extent of neonatal morbidity, and infant sex.

### 2.2. Stressor protocol

Our standardized ‘stressor’ protocol differed in its format at 1 month versus 6 and 12 months of infant age to be developmentally appropriate. Research Assistants (RA) were trained in each protocol, including oral and written instructions, practice of the protocol until proficient in a simulated setting with feedback, and then accompanying an expert Clinical Research Coordinator on at least 2 actual visits where the protocol was administered. Each protocol had distinct baseline, stressor, and post-stressor periods and was administered between 10 am and 12 pm to control for effects of circadian rhythm on cortisol levels. Mothers were present during the protocol but were only directly involved during the 6- and 12-month stressors.

#### 2.2.1. Stressor protocol at 1 month postnatal

This protocol involved a previously tested caregiving procedure shown to elicit a broad distribution of stress responses from infants in their first month of life (Weiss and Niemann, 2015). Three ECG sensors and a respiratory belt were placed on the infant’s chest. The ECG data was used for another component of the larger research project but was part of the protocol that occurred. After placement of the sensors and belt, infants were positioned on their back with their head to one side and swaddled in a blanket. Following this, no procedures or social interaction took place for 15 min before the baseline period began. At the start of the baseline period, the infant’s blanket was gently removed from the infant’s upper torso so the arms could move freely. The infant was then left undisturbed for 5 min with observation from a distance. At the end of the 5 min, the ‘stressor’ period began. At the start of the ‘stressor’ period, a saliva sample was acquired. The end of a *SalivaBio* infant swab was placed in the infant’s mouth between the cheek and bottom gum on the side facing downwards to collect pooled saliva. The swab was left in place for 5 min, being repositioned if needed to assure that the swab was saturated. After the swab was removed and placed in a storage tube, infants were positioned on their back. The RA then took the infant’s temperature, removed the diaper, performed peri-care, and applied a new diaper. Near the end of this caregiving period, the infant was again positioned on the back with head to one side. A second saliva sample was then acquired for DNA analyses unrelated to the aims described in this paper using the same procedure described above. The stressor protocol lasted 15 min. After collection of this sample, the infant was covered with a blanket and a ‘post-stressor’ period began during which the infant was left undisturbed for 5 min. Then the final saliva sample was acquired, using the same method as described previously. Total time from the onset of the caregiving stressor until we began collection of the final salivary sample was 20 min.

#### 2.2.2. Stressor protocol at 6 and 12 months postnatal

The ‘Repeated Still Face Paradigm’ (Mesman et al., 2009; Tronick, 2003) was used as the ‘stressor’ at 6 and 12 months of infant age. The ‘Still Face Paradigm’ is a well-established ethical ‘stressor’ shown to reliably elicit a physiologic stress response in infants (Lester et al., 2018; Ritz et al., 2020). During this procedure, mother and infant sat facing each other about 18–24 in. apart. Three ECG electrodes were placed on the chest of the infant who was given about 10 min to adjust to wearing them. Then the protocol was initiated. Prior to the start of the ‘Still Face Paradigm’, a saliva sample was acquired in the same manner as described for the 1-month protocol. The RA then cued the mother to begin each of 5 segments: baseline spontaneous play, 1st still face episode, another period of spontaneous play, 2nd still face episode, and final period of spontaneous play. In the spontaneous play segments, mother and infant interacted as they wished by talking, singing, or touching, but they could not use any toys and the infant could not be picked up. During the still face segments, the mother sat back in her chair and maintained a neutral expression. She continued to look at her baby’s face but was instructed not to talk, sing, smile, vocalize, or touch her baby (essentially to have a “poker” face). Each segment of the procedure lasted 2 min for a total of 10 min. At the end of this last spontaneous play episode, the infant’s EEG leads were removed by the mother and they began a period of free play. The purpose of the free play period was to support the infant’s transition to typical, usual interaction with the mother. The total time from the onset of the 1st still face episode until the end of the free play period was 20 min. The final saliva sample was acquired at the end of the free play period, using the same method as described previously.

#### 2.2.3. Procedures for managing excessive distress

Because our research involved infants, we developed a procedure (approved by our Institutional Review Board for Human Research) for managing any undue distress that might occur during the stressor protocols. If an infant became excessively distressed (e.g. strong ongoing crying with mottling or red face) during the stressor, we followed a series of steps which were discussed with the mother before the protocol began. The mother would briefly place her hand on the infant’s shoulder until the distress was minimized. During the stressor at 1 month postnatal only, mothers could alternately use their voice to soothe the infant. If this did not adequately address the distress, the mother would hold the infant briefly and then the protocol would be continued. If holding was necessary beyond 60 s and did not regulate the distress, the stressor protocol was discontinued and rescheduled for another day. During the stressor at 1 month postnatal, 16.7 % of the infants required help with their distress. At 6 months, 4.5 % required intervention, and 13.4 % at 12 months. We employed Mann-Whitney U tests to determine whether there were any differences in cortisol values for infants who deviated slightly from the standard protocol and those who did not. There were no significant differences between the two groups in any cortisol values. For baseline cortisol across the 3 timepoints, significance levels ranged from p = .37 to p = .77. For post-stressor cortisol, they ranged from p = .50 to p = .63, and, for cortisol reactivity, significance levels for differences between groups ranged from p = .23 to p = .86.

### 2.3. Measures

#### 2.3.1. Demographics

Information about women’s age, education, race and ethnicity, partnership and employment status, and financial security was acquired through a sociodemographic questionnaire. These data were used for description of the sample.

#### 2.3.2. Antenatal corticosteroid exposure and preterm birth status

Mothers’ receipt of betamethasone during pregnancy (the antenatal corticosteroid used at participating clinics) was identified through review of the medical record. The infant’s gestational age was also acquired from the medical record, with preterm birth status defined as less than 37 weeks’ gestation at birth.

#### 2.3.3. Infant salivary cortisol

Cortisol is a downstream measure of the body’s HPA axis response to stress. The first salivary sample (baseline resting state) and the final salivary sample (post-stressor) from each stressor protocol were used to assay average cortisol level of the infant prior to and after the stressor. To avoid contamination of cortisol samples, infants were not fed within 30 min prior to the RA’s arrival nor during the procedures. Biospecimens were stored at 20° Centigrade until they were sent to Salimetrics biochemical laboratory for analysis. At the lab, they were thawed to room temperature, vortexed, and centrifuged at 3000 RPM for 15 min prior to assay. Assays were performed with the Salimetric Salivary Cortisol Assay Kit, and high sensitivity salivary cortisol enzyme immunoassay (ELISA) used to analyze samples in duplicate. Sensitivity of the assay had a lower limit of .007 μg/dl, a standard curve ranging from 0.012 to 3.0 μg/dl, an average intra-assay coefficient of variation of 4.6, and an average inter-assay coefficient of variation of 6 %.

Infant cortisol level at resting state was the ug/dL value identified during the baseline period prior to the stressor while post-stressor cortisol was the ug/dL from the final sample after completion of the protocol. Infant cortisol reactivity was the difference between the ug/dL value at baseline and the value identified for the final post-stressor salivary sample. This change in cortisol levels indicates the magnitude of an infant’s response or amount of reactivity to the stressor.

#### 2.3.4. Covariates

Data on four covariates were acquired to examine their potential confounding effects and adjust for these in final analysis if needed. Covariates included obstetric medical risk, mothers’ perceived stress during pregnancy, and mothers’ endogenous cortisol levels during pregnancy (both average cortisol level and overall amount of cortisol secretion).

The Obstetric Medical Risk Index (Lobel et al., 2000) was used to extract data from the medical record regarding risks and complications related to pregnancy (e.g., placenta previa, polyhydramnios, cigarette smoking, anemia). The index is a validated and reliable tool, showing excellent predictive validity for adverse birth outcomes (Lobel et al., 2008). Perceived stress was examined through maternal completion of the Perceived Stress Scale during pregnancy and at 6 and 12 months postpartum (Cohen et al., 1983). The PSS measures the degree to which respondents feel their lives are unpredictable, uncontrollable, and overloaded with stressors over the four weeks prior to its completion. The questionnaire has well-established predictive validity and reliability across cultures and with varied populations of women. Women’s endogenous cortisol levels were assessed at the time of recruitment during the third trimester of pregnancy as well as at 6 and 12 months postpartum. At these timepoints, women provided four saliva samples each day across two consecutive days. They were taught how to collect saliva samples using the passive drool method by a member of the research team who verbally described and modeled the procedure. Women were also given written and pictorial instructions to use when providing salivary samples and were reminded to provide their samples by phone and text at the times they were scheduled for completion. They were asked to rinse their mouth with water 10 min prior to their sampling time. They drooled into a cryovial until 1 ml of saliva was accumulated as noted on the vial. Women provided samples when they awoke, 45 min after waking, around 4 pm, and just before sleep at night. They stored samples in their home freezer until they were either picked up by the RA or mailed to the research team. These cortisol samples were also assayed at the Salimetrics biochemical laboratory using procedures described earlier for infant cortisol samples. Two cortisol scores were developed from women’s saliva samples: their average cortisol level across the 8 sampling times over a two-day period, and the mean area under the curve (AUC_G_) for the 2-day samples. AUC_G_ measured the amount of total cortisol output across the day, considering the difference between individual cortisol samples and the time between each sampling period. We calculated AUC_G_ with the trapezoidal formula from Pruessner et al. (2003).

#### 2.3.5. Data analyses

Descriptive statistics were used to characterize the sample. We used mixed effects multilevel regression to determine if fetal AC exposure during gestation was associated with infant resting state cortisol level, cortisol reactivity to a stressor, or post-stressor cortisol level at 1 month, 6 months, or 12 months postnatal. We employed Full Information Maximum Likelihood (FIML) methods to achieve unbiased estimates of the effects over time for data missing at random. A bias-corrected nonparametric bootstrap was employed with 1000 replications to accommodate non-normality. We employed nonparametric bootstrapping since standard approaches such as winsorizing of outliers and log transformation of cortisol variables were insufficient to correct for lack of normality in cortisol distributions. Excluding extreme values solely due to their extremeness can distort results by removing important information about variability inherent in the sample. Regardless, their removal did not effectively address skew for some variables. Bootstrapping techniques enable retention of all values when log transformations do not effectively address a skewed distribution. Thus, bootstrapping allowed us to capture the full variability of our dataset without violating assumptions of normality and to interpret results on the original scale. A Bonferroni adjustment to the confidence intervals (98.33 %) was also applied to account for multiple comparisons. Initial Spearman correlations were examined between each infant cortisol metric and the four continuous covariates: obstetric medical risk, maternal pregnancy stress, and maternal endogenous cortisol during pregnancy (both average cortisol level and overall amount of cortisol secretion). Covariates showing a significant relationship at p = .05 or less were included in the final regression models. In addition, we assessed the potential for infant sex differences in preliminary models, computing its main effect on cortisol levels and cross-level interactions between AC exposure and sex. To test Aim 2, we examined a cross-level interaction in the models between AC exposure and preterm birth status. We carried out statistical analyses with Stata 16 (Stata Corp, 2019). We evaluated all tests of significance with a two-sided alpha of 0.05.

## 3. Results

### 3.1. Sample characteristics

Data from 181 mothers and infants were included in this analysis. Descriptive statistics for the sample are presented in Table 1. Mothers were an average of 33.11 years old and represented diverse racial and ethnic backgrounds. Just over half of the mothers identified their race as White/European-American (51 %), while 23 % identified as African American/Black, 19 % as Asian American, 2 % as Hawaiian/Pacific Islander or Native American, and about 4 % were unknown. About 24 % of mothers reported Hispanic/Latina ethnicity. Mothers in the sample were more likely to be well-educated and report relatively high annual household income levels. Infants had a mean gestational age of 36.6 weeks (range: 27.3 – 43), 51 % of the sample were born preterm, and 39 % had been exposed to AC during gestation. 78 % (n = 141) of infants who were exposed to AC were born preterm. Sex was evenly distributed between males and females. Diversity in infant race and ethnicity was distributed similarly to the mothers.

### 3.2. Infant cortisol over the three timepoints

We examined the stability of infant cortisol levels from 1 month to 12 months of infant age. First, we calculated Intraclass Correlation Coefficients (ICC) for infant baseline and post-stressor cortisol as a measure of intra-individual stability over 1, 6 and 12 months of infant age. The ICC for baseline cortisol was .33 (SE:.06; 95 % CI:.2272,0.4613). For post-stressor cortisol, the ICC was .26 (SE:.06; 95 % CI:.1572,0.4003). Cortisol reactivity had an ICC of .06 (SE:.04; CI:.0136,0.2574). All coefficients indicate a high level of cortisol variability for infants across time, likely due to changes in environmental exposures and effects of development. We also used Friedman’s non-parametric test for repeated measures to assess stability of the entire sample’s cortisol data over time. After Bonferroni corrections for multiple testing, differences in baseline cortisol (χ^2^ = 3.47 (df=2), p = .17), post-stressor cortisol (χ^2^ = 6.86 (df=2), p = .03), and cortisol reactivity (χ^2^ = 0.946 (df=2), p = .62) were not significant across the 3 timepoints. Pairwise comparisons between timepoints did indicate trends toward a significant difference between 1 and 6 months of age for both level of baseline cortisol (z = 1.82, p = .07) and post-stressor cortisol (z = 1.79, p = .07) but not for cortisol reactivity. Table 2 provides data on cortisol values for baseline, post-stressor and reactivity metrics at the 3 developmental timepoints for infants exposed and not exposed to AC.

### 3.3. Infant response to the stressor protocols

For the sample as a whole, the stressor protocol at 1 month postnatal elicited a significant mean increase of .197 (0.08) from baseline (0.578 μg/dl) to post-stressor (0.775 μg/dl) based on *t*-tests estimated with the nonparametric bootstrap (95 % BC CI: 0.0578,0.3769). For the stressor protocol at 6 months postnatal, we did not find a significant increase from baseline to post-stressor for the entire sample of infants (95 % BC CI: 0.0676,0.2135). On average, infants increased by .062 (0.07) from baseline cortisol (0.587 μg/dl) to post-stressor cortisol (0.649 μg/dl). In contrast, the 12-month stressor protocol did show a significant mean increase of .259 (0.15) μg/dl (95 % BC CI: 0.0320,0.6439) from baseline (0.437 μg/dl) to post-stressor (0.697 μg/dl). We ran all bootstraps with 5000 repetitions.

### 3.4. Preliminary analyses of covariates

Preliminary correlations between maternal prenatal covariates and infant cortisol variables are shown in Table 3. Only women’s ‘Area Under the Curve’ (AUC_G_) for their cortisol during pregnancy was significantly associated with any infant cortisol variable. Infants whose mothers had a higher AUC_G_ during pregnancy had a higher baseline resting state cortisol level as well as a higher post-stressor cortisol level at 12 months of age. Based on this relationship, we did include AUC_G_ in our multilevel regression modeling to examine the aims. However, AUC_G_ had no relationship to any of the infant cortisol metrics when considered with other variables. For example, the direct effect of AUC_G_ on infant resting state cortisol was B = 0.00 (CI: −0.0046,0.0147). The moderating effect of AUC_G_ on the relationship between AC exposure and infant resting state cortisol was B = 0.00 (CI: −0.0211,0.0041). Similarly, the direct effect of AUC_G_ on infant post-stressor cortisol was B = 0.03 (CI: −0.0139,0.0767). The moderating effect of AUC_G_ on the relationship between AC exposure and infant post-stressor cortisol was B = 0.01 (CI: −0.0801,0.0143). Consequently, AUC_G_ was not included in the final models.

### 3.5. Testing of the aims

**Fig. 1.**
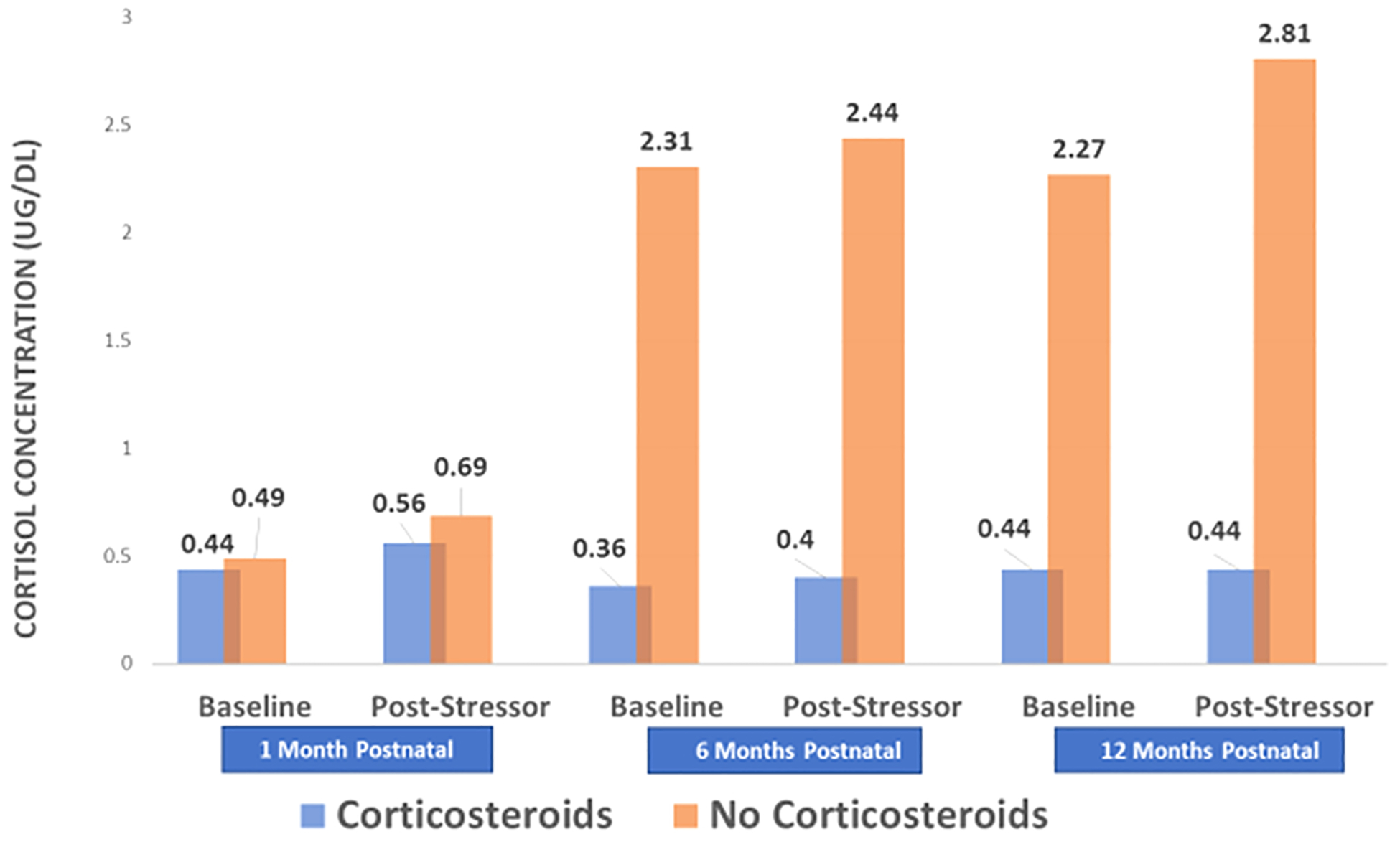
Infant cortisol levels before and after a stressor at 1, 6 and 12 Months of Age. Note: Baseline cortisol specimens were acquired after a 15-minute period when the infant had no procedures or unusual social interaction and immediately prior to onset of the stressor. Collection of post-stressor cortisol specimens began 20 min after the onset of the stressor.

Results of the regressions for infant resting state (baseline) cortisol and cortisol after the stressor (post-stressor) are presented in Table 4. AC exposure was significantly associated with mean resting state cortisol of infants across the 12 months (B = −2.47, BC CI:− 3.691, −0.0484). Adjusting for preterm birth status, infants whose mothers received AC during pregnancy had significantly lower average resting state cortisol levels across the first year of life (x‾ = 0.52) than infants whose mothers did not receive AC (x‾ = 2.91). There were no unique interactions of AC exposure with any of the 3 age time points (1, 6, or 12 months) at which infant resting state cortisol was assessed. Fig. 1 highlights the differences in basal and post-stressor cortisol levels across the three timepoints. In contrast to AC, preterm birth was not associated with the infant’s resting state/basal cortisol level and did not moderate the relationship between AC exposure and cortisol level.

Findings for post-stressor infant cortisol support the same significant association with AC exposure as for infant resting state (B = −2.51, BC CI: −4.283, −0.4276). Infants whose mothers received AC during pregnancy had significantly lower post-stressor cortisol levels across the first year of life (x‾ = 0.43) than infants whose mothers did not receive AC (x‾ = 3.09). There was no effect of preterm birth status on infants’ post-stressor cortisol (neither a direct nor moderating effect).

For cortisol reactivity, there was no association of either AC exposure or preterm birth to infant cortisol reactivity in response to a stressor (Table 5). The reactivity values shown in Table 2 suggest there may have been a difference between infants exposed to AC (x‾ = 0.01) and those not exposed (x‾ = 0.54) in their reactivity at 12 months. However, the bootstrapped correction for bias indicated a lack of significance between groups based upon 98.33 % CI (B = −0.81, CI: −1.88, −0.27).

In examining the potential role of infant sex, we found no significant differences between male and female infants in any of the cortisol variables across the 3 timepoints, including average baseline cortisol (B = −0.53; CI: −2.29, 1.23), post-stressor cortisol (B = −0.71; CI: −2.62, 1.19), or cortisol reactivity (B = 0.22; CI: −0.45, 0.89). In addition, infant sex did not moderate the relationship between AC exposure and either baseline (B = 0.27; CI: 1.69, 1.15), post-stressor (B = −0.47; CI: −1.19, 2.12), or cortisol reactivity levels (B = −0.25; CI: −1.22, 0.72).

## 4. Discussion

### 4.1. AC and basal cortisol levels

Our results indicate that infants who were exposed to AC had lower resting cortisol levels and dampened cortisol levels after exposure to a stressor. These responses occurred at 1, 6, and 12 months of age, indicating a persistent hypo-arousal of their HPA axis throughout the first year of life compared with infants who were not exposed to AC. Our findings support previous research linking AC exposure to suppression of the HPA axis in the early postnatal period and provide new evidence that this effect may persist longer than previously known. Changes observed in exposed infants may result from fetal adaptations that influenced development of their HPA axis. In typical HPA axis development, the placenta protects the fetus by modulating exposure to maternal cortisol via the 11βHSD2 enzyme, which oxidizes cortisol into its inactive metabolite, cortisone. However, synthetic GC such as betamethasone and dexamethasone are not readily metabolized by 11βHSD2 (Reynolds, 2013; Tegethoff et al., 2009; Waffarn and Davis, 2012). They cross the placental barrier (Chatuphonprasert et al., 2018), leaving the fetus exposed to their effects. This could result in excessive glucocorticoid exposure at a critical juncture in development, precipitating alterations in the fetal HPA axis that yield greater suppression of hormonal secretion.

Research using animal models has shown that AC administration can have significant effects on the developing HPA axis (Kapoor et al., 2008). The fetal brain contains high levels of glucocorticoid receptors (GR) by the 3rd trimester, with some of the highest levels in areas of the brain that control secretion of CRH and ACTH production (Asztalos, 2012). Kapoor et al. (2008) note that prenatal exposure to AC leads to permanent changes in the expression of GRs and mineralocorticoid receptors (MRs), resulting in altered negative feedback sensitivity and altered set points for HPA function. The fetus is protected from exposure to elevated levels of cortisol via a negative feedback loop activated by GRs and MRs throughout the HPA axis. There is growing evidence that exposure to exogenous GC can lead to an alteration of these receptors in human infants (Chang, 2014; Davis et al., 2006; Moisiadis and Matthews, 2014; Tegethoff et al., 2009; Waffarn and Davis, 2012). Our finding that cortisol hypoactivation persists throughout the first year of life indicates that the effects of AC exposure on HPA axis function of human infants may last longer than previously shown.

Previous evidence demonstrates that exposure to prenatal stress may also impact fetal HPA axis development, through exposure to elevated levels of the mother’s endogenous cortisol (McGowan and Matthews, 2018). Our findings that infant cortisol suppression was significant, even after controlling for mothers’ own endogenous cortisol levels, indicate that exogenous glucocorticoid exposure during the third trimester could be more salient in programming infant stress regulation than exposure to mothers’ stress hormones. These results may help in clarifying previous research that has noted difficulty untangling the impact of mothers’ own stress from the effect of AC (Hwang et al., 2019; McGowan and Matthews, 2018).

### 4.2. AC and cortisol reactivity

Despite our findings that AC exposure significantly affected resting and post-stressor cortisol levels, this effect was not true for cortisol reactivity at any assessment time point. This could suggest that perturbations to fetal HPA axis development induced by AC likely targeted basal or tonic levels of activation rather than mechanisms underlying responsiveness to a specific stressor. Different types of exposure can be linked to distinctly different alterations in cortisol regulation (Epstein et al., 2021). Based on their synthesis of research, Henckens et al. (2016) propose that basal levels of cortisol and the stress-induced cortisol response involve different patterns of corticosteroid receptor activation and likely have differential neural correlates. Animal studies suggest that basal cortisol levels mainly involve activation of nuclear MRs which are more targeted to general neuronal homeostasis in readiness for stress, while the stress-induced rise in cortisol involves activation of low-affinity MRs and GRs in the cell membrane that potentiate rapid response to stressors and arousal (Groeneweg et al., 2012).

Alternatively, contrasting patterns of cortisol reactivity among infants may have masked any effects of AC on our sample’s overall response to the stressor. In a review and meta-analysis of infant cortisol reactivity, Provenzi et al. (2016) found that infants in the first year of life show varied patterns of reactivity to stressor protocols, specifically the ‘Still Face Paradigm’. Cortisol levels of some infants increased when exposed to the stressor while others decreased and still others remained stable. Better understanding how exposure to AC may influence infants with distinct patterns of cortisol reactivity is an essential area for future research.

### 4.3. Effects of preterm birth

To our knowledge, this was the first study to examine the moderating effects of prematurity on AC exposure in a large sample that included term infants. Preterm birth status had no direct or interaction effects with AC exposure on infant resting state, cortisol reactivity, or post-stressor cortisol levels at any assessment time point. The lack of any moderating effect is congruent with findings from previous studies of preterm infants that gestational age does not moderate AC effects on cortisol (Ashwood et al., 2006; Karlsson et al., 2000), and extends the evidence to include a comparison with term infants. However, the absence of a direct effect of prematurity conflicts with findings in a previous study indicating that gestational age was a stronger predictor of cortisol level than AC exposure (Ng et al., 2019), although infants in their sample were younger than 28 weeks while premature infants in our sample were born around 34 weeks on average. Very young gestational age is accompanied by a less well-developed neuroendocrine system and greater likelihood of being exposed to stressful health care procedures for which an immature HPA axis is ill-equipped (Lammertink et al., 2021; Masumoto et al., 2019). It is possible that the interaction effects of gestational age with AC could be more pronounced for infants born extremely preterm.

### 4.4. Implications

Our results support previous research that AC exposure may have adverse consequences for fetal and infant health, calling attention to the need for careful consideration when prescribing AC and for administration guidelines that maximize benefits while minimizing risks for the fetus. More robust research is needed to examine the interaction between exogenous and endogenous glucocorticoid exposure and their effects on stress regulation of children, avoiding attribution of fault to mothers by better accounting for external influences on fetal development of adrenocortical function. While our results support previous evidence of glucocorticoid programming effects on fetal HPA axis development (Moisiadis and Matthews, 2014), little is known about the complex mechanisms underlying these effects. In particular, it is essential to examine differential mechanisms that may explain why tonic/basal cortisol is more affected by AC than is cortisol reactivity that is induced by stressors. Evaluating epigenetic alterations that may drive patterns of cortisol secretion, negative feedback processes, and receptor binding across the HPA axis may have particular merit.

Our findings provide new evidence that the impact of AC exposure on the HPA axis persists throughout the first year of life, but future research is needed to explore these associations in toddler and early childhood age groups where evidence remains sparse. While cortisol levels provide physiologic evidence of the impact of AC, studies need to relate alterations of the HPA axis to potential effects on infant emotional regulation, which has been closely linked to psychopathology in later childhood (Cook et al., 2019). A better understanding of this pathway could inform neonatal or pediatric interventions to mitigate adverse mental health outcomes, such as those that apply emotional regulation strategies to improve cortisol function.

### 4.5. Limitations and strengths

Findings should be interpreted in the context of the study’s limitations and strengths. Our sample consisted of mothers receiving prenatal care, with relatively high levels of education and income; generaliz-ability of our findings is limited to similar populations. The majority of preterm infants in our sample was late preterm, which may have limited our ability to see interaction effects for gestational age that might be more pronounced in younger and more vulnerable age groups. Finally, we performed the Still Face Paradigm on only one occasion at each of the 6- and 12-month time periods; multiple assessments would have increased the reliability of results.

However, the size as well as racial and ethnic diversity of our sample are strengths; many of the previous studies examining AC and infant cortisol involve smaller, more homogenous European populations. Experiences of stress and the circumstances of preterm birth are known to vary among diverse populations in the U.S. (Almeida et al., 2018); therefore, it is important that studies include varied representations. The study was also strengthened by our comparison to term infants when considering the overall moderating effect of preterm birth status.

## 5. Conclusions

AC may significantly reduce morbidity and mortality for infants delivered preterm; but the potential risks associated with their use must be considered along with their benefits during clinical decision making. Our results indicate a state of dampened HPA activation and cortisol hypo-arousal that persists across the first year of life among infants who were exposed to corticosteroids in utero. More research is needed to understand critical developmental periods in HPA axis and stress regulation during which interventions may be most beneficial.

## Figures and Tables

**Table 1:** Characteristics of the sample (N = 181).

Maternal Age (Years) - mean (SD)		33.11 (5.98)
Infant Gestational Age (Weeks) - mean (SD)		36.6 (2.76)
AC Exposure – n (%)		
Non–exposed		110 (61)
Exposed		71 (39)
Infant Preterm Birth Status – n (%)		
Preterm		92 (51)
Term		89 (49)
Infant Sex Assigned at Birth – n (%)		
Male		91 (50)
Female		90 (50)
Mother–reported Hispanic/Latinx Ethnicity – n (%)		
Mother		43 (24)
Baby		45 (25)
Mother–Reported Race – n (%)		
Euro–American/White –	Mother	92 (51)
	Baby	89 (49)
African American/Black –	Mother	41 (23)
	Baby	41 (23)
Asian American –	Mother	34 (19)
	Baby	41 (23)
Hawaiian/PI/Native American –	Mother	4 (2.2)
	Baby	5 (3.6)
Unknown –	Mother	8 (4.4)
	Baby	1 (0.7)
Mother’s Highest Level of Education – n (%)		
High school or less		40 (22)
Some college		37 (20)
College graduate		29 (16)
Graduate degree		55 (30)
Unknown		20 (11)
Income – n (%)		
< 20k		46 (27)
21–50k		20 (11)
51–100k		7 (3)
101–150k		13 (8)
Above 150k		67 (36)
Unknown		28 (15)

**Table 2:** Cortisol baseline, reactivity and post-stressor values (ug/dL) at 1, 6 and 12 months postnatal for infants exposed to antenatal corticosteroids and infants not exposed.

	Exposed		Not Exposed	
	Mean (SE)	Range	Mean (SE)	Range
Infant Cortisol at 1 Month Postnatal				
Baseline	44 (0.11)	0.06–3.28	0.4 (0.14)	0.07 – 5.18
Reactivity	0.12 (0.05)	−0.93–0.47	0.20 (0.18)	−7.21 – 3.60
Post-Stressor	0.56 (0.19)	0.07–3.83	0.69 (0.14)	0.06 – 8.82
Infant Cortisol at 6 Months Postnatal				
Baseline	0.36 (0.10)	0.05 – 5.51	2.31 (0.95)	0.08 – 7.02
Reactivity	0.04 (0.06)	−0.80 –0.47	0.13 (0.44)	−5.40 – 3.62
Post-Stressor	0.40 (0.07)	0.09 – 3.71	2.44 (0.56)	0.08 – 9.33
Infant Cortisol at 12 Months Postnatal				
Baseline	0.44 (0.04)	0.04 – 2.00	2.27 (0.84)	0.05 – 5.85
Reactivity	01 (0.13)	−1.71 – 4.55	0.54 (0.31)	−0.53 – 9.63
Post-Stressor	0.44 (0.26)	0.04 – 6.54	2.81 (0.97)	0.05 – 7.18

**Table 3:** Spearman correlations between maternal prenatal covariates and infant cortisol metrics at 1, 6, and 12 months postnatal.

	Obstetric Risk	Maternal Stress	Maternal Cortisol Average Level	AUC_G_

Infant Cortisol at 1 Month Postnatal				
Baseline	0.00	−0.06	−0.04	−0.01
Reactivity	−0.01	−0.07	0.07	0.15
Post-Stressor	−0.01	−0.00	−0.08	−0.11
			
Infant Cortisol at 6 Months Postnatal				
Baseline	0.05	0.07	0.01	0.08
Reactivity	0.16	0.07	0.16	0.07
Post-Stressor	−0.08	−0.02	−0.00	0.14
			
Infant Cortisol at 12 Months Postnatal				
Baseline	0.00	0.04	0.09	0.26 *
Reactivity	−0.16	−0.03	−0.01	0.02
Post-Stressor	−0.08	−0.01	0.18	0.31 * *
			

AUC_G_ = Area Under the Curve;

* p = .05;

* * p = .01

**Table 4:** Multilevel model for relationships of antenatal corticosteroid (AC) exposure and preterm birth to infant means for baseline and post-stressor cortisol.

	Baseline Cortisol			Post-Stressor Cortisol		
	98.33 % Bootstrap Corrected CI			98.33 % Bootstrap Corrected CI		
	Estimate	Lower Bound	Upper Bound	Estimate	Lower Bound	Upper Bound
Model 1						
6 Months of Age*	1.12	−0.1222	2.406	0.99	−0.2615	2.208
12 Months of Age	1.13	0.2027	2.816	1.20	−0.2871	2.913
Model 2						
AC Exposure	−2.47	−3.691	−0.0484 * *	−2.51	−4.283	−0.4276 * *
Preterm Birth	1.75	−0.6105	3.142	1.39	−0.6837	3.241
Model 3						
AC Exposure × Preterm Birth	−3.85	−10.962	0.7799	−3.47	−11.485	1.197

Non-parametric bootstrapped bias-corrected confidence intervals (5000 repetitions). If zero is in the interval, the estimate is not significant. Standard errors and t or z statistics are not relevant for the nonparametric bootstrap.

* Reference point is 1 month of life;

* * Significant

**Table 5:** Multilevel model for relationships of antenatal corticosteroid (AC) exposure and preterm birth to infant mean cortisol reactivity in response to a stressor.

		98.33 % Bootstrap Corrected Confidence Interval	
	Estimate	Lower Bound	Upper Bound
Model 1			
6 Months of Age*	− 0.13	− 1.43	1.16
12 Months of Age	0.99	0.06	1.93 * *
Model 2			
AC Exposure	0.19	−0.51	0.89
Preterm Birth	0.29	−1.13	1.72
Model 3			
AC Exposure × Preterm Birth	−0.19	−1.75	1.36

Non-parametric bootstrapped bias-corrected confidence intervals (5000 repetitions). If zero is in the interval, the estimate is not significant. Standard errors and t or z statistics are not relevant for the nonparametric bootstrap.

* Reference point is 1 month of life;

* * Significant.

## References

[R1] Alexander N, Rosenlöcher F, Stalder T, Linke J, Distler W, Morgner J, Kirschbaum C, 2012. Impact of antenatal synthetic glucocorticoid exposure on endocrine stress reactivity in term-born children (Oct)). J. Clin. Endocrinol. Metab 97 (10), 3538–3544. 10.1210/jc.2012-1970. 22869608

[R2] Almeida J, Bécares L, Erbetta K, Bettegowda VR, Ahluwalia IB, 2018. Racial/ethnic inequities in low birth weight and preterm birth: the role of multiple forms of stress (Aug).). Matern Child Health J 22 (8), 1154–1163. 10.1007/s10995-018-2500-7. 29442278 PMC10998226

[R3] Ashwood PJ, Crowther CA, Willson KJ, Haslam RR, Kennaway DJ, Hiller JE, Robinson JS, 2006. Neonatal adrenal function after repeat dose prenatal corticosteroids: a randomized controlled trial (Mar).). Am. J. Obstet. Gynecol 194 (3), 861–867. 10.1016/j.ajog.2005.08.063. 16522426

[R4] Asztalos E, 2012. Antenatal corticosteroids: a risk factor for the development of chronic disease (2012, 2012/03/05). J. Nutr. Metab, 930591. 10.1155/2012/930591. 22523677 PMC3317130

[R5] Buyukkayhan D, Ozturk MA, Kurtoglu S, Koklu E, Yikilmaz A, 2009. Effect of antenatal betamethasone use on adrenal gland size and endogenous cortisol and 17-hydroxyprogesterone in preterm neonates (Nov)). J. Pedia Endocrinol. Metab 22 (11), 1027–1031. 10.1515/jpem.2009.22.11.1027. 20101888

[R6] Cabral DM, Antonini SR, Custódio RJ, Martinelli CE Jr., da Silva CA, 2013. Measurement of salivary cortisol as a marker of stress in newborns in a neonatal intensive care unit. Horm. Res Paediatr 79 (6), 373–378. 10.1159/000351942. 23796826

[R7] Chang YP, 2014. Evidence for adverse effect of perinatal glucocorticoid use on the developing brain (Mar)). Korean J. Pedia 57 (3), 101–109. 10.3345/kjp.2014.57.3.101. PMC4000755 24778691

[R8] Chatuphonprasert W, Jarukamjorn K, Ellinger I, 2018. Physiology and pathophysiology of steroid biosynthesis, transport and metabolism in the human placenta. Front Pharm 9, 1027. 10.3389/fphar.2018.01027. PMC6144938 30258364

[R9] Cohen S, Kamarck T, Mermelstein R, 1983. A global measure of perceived stress. J. Health Soc. Behav 385–396. 6668417

[R10] Cook F, Giallo R, Hiscock H, Mensah F, Sanchez K, Reilly S, 2019. Infant regulation and child mental health concerns: a longitudinal study. Pediatrics 143 (3). 10.1542/peds.2018-0977. 30737245

[R11] Dalziel SR, Walker NK, Parag V, Mantell C, Rea HH, Rodgers A, Harding JE, 2005. Cardiovascular risk factors after antenatal exposure to betamethasone: 30-year follow-up of a randomised controlled trial. May 28-Jun 3). Lancet 365 (9474), 1856–1862. 10.1016/s0140-6736(05)66617-2. 15924982

[R12] Davis EP, Townsend EL, Gunnar MR, Georgieff MK, Guiang SF, Ciffuentes RF, Lussky RC, 2004. Effects of prenatal betamethasone exposure on regulation of stress physiology in healthy premature infants (Sep)). Psychoneuroendocrinology 29 (8), 1028–1036. 10.1016/j.psyneuen.2003.10.005. 15219654

[R13] Davis EP, Townsend EL, Gunnar MR, Guiang SF, Lussky RC, Cifuentes RF, Georgieff MK, 2006. Antenatal betamethasone treatment has a persisting influence on infant HPA axis regulation (Mar)). J. Perinatol 26 (3), 147–153. 10.1038/sj.jp.7211447. 16467857

[R14] Davis EP, Waffarn F, Sandman CA, 2011. Prenatal treatment with glucocorticoids sensitizes the hpa axis response to stress among full-term infants (Mar)). Dev. Psychobiol 53 (2), 175–183. 10.1002/dev.20510. 21298632 PMC10486314

[R15] Epstein CM, Houfek JF, Rice MJ, Weiss SJ, 2021. Integrative review of early life adversity and cortisol regulation in pregnancy (May)). J. Obstet. Gynecol. Neonatal Nurs 50 (3), 242–255. 10.1016/j.jogn.2020.12.006. PMC8113070 33524324

[R16] Erni K, Shaqiri-Emini L, La Marca R, Zimmermann R, Ehlert U, 2012. Psychobiological effects of prenatal glucocorticoid exposure in 10-year-old-children. Front Psychiatry 3, 104. 10.3389/fpsyt.2012.00104. 23233841 PMC3517968

[R17] Glover V, Miles R, Matta S, Modi N, Stevenson J, 2005. Glucocorticoid exposure in preterm babies predicts saliva cortisol response to immunization at 4 months (Dec)). Pedia Res 58 (6), 1233–1237. 10.1203/01.pdr.0000185132.38209.73. 16306199

[R18] Gover A, Brummelte S, Synnes AR, Miller SP, Brant R, Weinberg J, Grunau RE, 2012. Single course of antenatal steroids did not alter cortisol in preterm infants up to 18 months (Jun)). Acta Paediatr 101 (6), 604–608. 10.1111/j.1651-2227.2012.02629.x. 22313364 PMC4833441

[R19] Groeneweg FL, Karst H, de Kloet ER, Jöels M, 2012,. Mineralocorticoid and glucocorticoid receptors at the neuronal membrane, regulators of nongenomic corticosteroid signalling. Mol. Cell Endocrinol 350 (2), 299–309. 10.1016/j.mce.2011.06.020. 21736918

[R20] Henckens MJ, Klumpers F, Everaerd D, Kooijman SC, van Wingen GA, Fernández G, 2016. Interindividual differences in stress sensitivity: basal and stress-induced cortisol levels differentially predict neural vigilance processing under stress (Apr)). Soc. Cogn. Affect Neurosci 11 (4), 663–673. 10.1093/scan/nsv149. 26668010 PMC4814795

[R21] Hwang JH, Lee BS, Kim CY, Jung E, Kim EA, Kim KS, 2019. Basal serum cortisol concentration in very low birth weight infants (Dec)). Pedia Neonatol 60 (6), 648–653. 10.1016/j.pedneo.2019.03.003. 30962158

[R22] Ilg L, Kirschbaum C, Li SC, Rosenlöcher F, Miller R, Alexander N, 2019. Persistent effects of antenatal synthetic glucocorticoids on endocrine stress reactivity from childhood to adolescence. Mar 1). J. Clin. Endocrinol. Metab 104 (3), 827–834. 10.1210/jc.2018-01566. 30285119

[R23] Kajantie E, Raivio T, Jänne OA, Hovi P, Dunkel L, Andersson S, 2004. Circulating glucocorticoid bioactivity in the preterm newborn after antenatal betamethasone treatment (Aug)). J. Clin. Endocrinol. Metab 89 (8), 3999–4003. 10.1210/jc.2004-0013. 15292340

[R24] Kapoor A, Petropoulos S, Matthews SG, 2008. Fetal programming of hypothalamic–pituitary–adrenal (HPA) axis function and behavior by synthetic glucocorticoids, 2008/03/14/). Brain Res. Rev 57 (2), 586–595. 10.1016/j.brainresrev.2007.06.013. 17716742

[R25] Karlsson R, Kallio J, Toppari J, Scheinin M, Kero P, 2000. Antenatal and early postnatal dexamethasone treatment decreases cortisol secretion in preterm infants. Horm. Res 53 (4), 170–176. 10.1159/000023563. 11044800

[R26] Kemp MW, Jobe AH, Usuda H, Nathanielsz PW, Li C, Kuo A, Huber HF, Clarke GD, Saito M, Newnham JP, Stock SJ, 2018. Efficacy and safety of antenatal steroids. Oct 1). Am. J. Physiol. Regul. Integr. Comp. Physiol 315 (4), R825–r839. 10.1152/ajpregu.00193.2017. 29641233 PMC11961112

[R27] Lammertink F, Vinkers CH, Tataranno ML, Benders M, 2020. Premature birth and developmental programming: mechanisms of resilience and vulnerability. Front Psychiatry 11, 531571. 10.3389/fpsyt.2020.531571. 33488409 PMC7820177

[R28] Lester B, Conradt E, Lagasse L, Tronick E, Padbury J, Marsit C, 2018. Epigenetic programming by maternal behavior in the human infant. Pediatrics 142, e20171890. 10.1542/peds.2017-1890. 30257918 PMC6192679

[R29] Lobel M, DeVincent CJ, Kaminer A, Meyer BA, 2000. The impact of prenatal maternal stress and optimistic disposition on birth outcomes in medically high-risk women. Health Psychol 19 (6), 544–553. 10.1037/0278-6133.19.6.544. 11129357

[R30] Lobel M, Cannella DL, Graham JE, DeVincent C, Schneider J, Meyer BA, 2008. Pregnancy-specific stress, prenatal health behaviors, and birth outcomes. Health Psychol 27 (5), 604–615. 10.1037/a0013242. 18823187

[R31] Masumoto K, Tagawa N, Kobayashi Y, Kusuda S, 2019. Cortisol production in preterm infants with or without late-onset adrenal insufficiency of prematurity: a prospective observational study. Pediatr. Neonatol 60 (5), 504–511. 30670349 10.1016/j.pedneo.2018.12.001

[R32] McGoldrick E, Stewart F, Parker R, Dalziel SR, 2020. Antenatal corticosteroids for accelerating fetal lung maturation for women at risk of preterm birth. Cochrane Database Syst. Rev 12 (12), Cd004454. 10.1002/14651858.CD004454.pub4. 33368142 PMC8094626

[R33] McGowan PO, Matthews SG, 2018. Prenatal stress, glucocorticoids, and developmental programming of the stress response. Endocrinology 159 (1), 69–82. 10.1210/en.2017-00896. 29136116

[R34] Mesman J, van Ijzendoorn MH, Bakermans-Kranenburg MJ, 2009. The many faces of the still-face paradigm: a review and meta-analysis. Dev. Rev 29 (2), 120–162. 10.1016/j.dr.2009.02.001.

[R35] Miller NM, Williamson C, Fisk NM, Glover V, 2004. Infant cortisol response after prolonged antenatal prednisolone treatment (Dec). Bjog 111 (12), 1471–1474. 10.1111/j.1471-0528.2004.00288.x. 15663140

[R36] Moisiadis VG, Matthews SG, 2014. Glucocorticoids and fetal programming part 1: outcomes (Jul).). Nat. Rev. Endocrinol 10 (7), 391–402. 10.1038/nrendo.2014.73. 24863382

[R37] Ng SM, Ogundiya A, Didi M, Turner MA, 2019. Adrenal function of extremely premature infants in the first 5 days after birth. Apr 24 J. Pedia Endocrinol. Metab 32 (4), 363–367. 10.1515/jpem-2018-0417. 30849046

[R38] Niwa F, Kawai M, Kanazawa H, Okanoya K, Myowa M, 2020. The development of the hypothalamus-pituitary-adrenal axis during infancy may be affected by antenatal glucocorticoid therapy. J. Neonatal Perinat. Med 13 (1), 55–61. 10.3233/npm-180040. 31609703

[R39] Nykänen P, Raivio T, Heinonen K, Janne OA, Voutilainen R, 2007. Circulating glucocorticoid bioactivity and serum cortisol concentrations in premature infants: the influence of exogenous glucocorticoids and clinical factors (May). Eur. J. Endocrinol 156 (5), 577–583. 10.1530/eje-06-0672. 17468194

[R40] Provenzi L, Giusti L, Montirosso R, 2016. Do infants exhibit significant cortisol reactivity to the Face-to-Face Still-Face paradigm? A narrative review and meta-analysis. Dev. Rev 42, 34–55.

[R41] Pruessner JC, Kirschbaum C, Meinlschmid G, Hellhammer DH, 2003. Two formulas for computation of the area under the curve represent measures of total hormone concentration versus time-dependent change (Oct). Psychoneuroendocrinology 28 (7), 916–931. 10.1016/s0306-4530(02)00108-7. 12892658

[R42] Reynolds RM, 2013. Programming effects of glucocorticoids (Sep). Clin. Obstet. Gynecol 56 (3), 602–609. 10.1097/GRF.0b013e31829939f7. 23722920

[R43] Ritz T, Schulz SM, Rosenfield D, Wright RJ, Bosquet Enlow M, 2020. Cardiac sympathetic activation and parasympathetic withdrawal during psychosocial stress exposure in 6-month-old infants. Psychophysiology 57 (12), e13673. 10.1111/psyp.13673. 33048371 PMC8548071

[R44] Roberts D, Brown J, Medley N, Dalziel SR, 2017. Antenatal corticosteroids for accelerating fetal lung maturation for women at risk of preterm birth. Mar 21). Cochrane Database Syst. Rev 3 (3), Cd004454. 10.1002/14651858.CD004454.pub3. 28321847 PMC6464568

[R45] Schäffer L, Luzi F, Burkhardt T, Rauh M, Beinder E, 2009. Antenatal betamethasone administration alters stress physiology in healthy neonates (May). Obstet. Gynecol 113 (5), 1082–1088. 10.1097/AOG.0b013e3181a1f0e6. 19384124

[R46] Society for Maternal-Fetal Medicine (SMFM), 2016. Implementation of the use of antenatal corticosteroids in the late preterm birth period in women at risk for preterm delivery (Aug). Am. J. Obstet. Gynecol 215 (2), B13–B15. 10.1016/j.ajog.2016.03.013. 26992737

[R47] Tegethoff M, Pryce C, Meinlschmidt G, 2009. Effects of intrauterine exposure to synthetic glucocorticoids on fetal, newborn, and infant hypothalamic-pituitary-adrenal axis function in humans: a systematic review (Dec). Endocr. Rev 30 (7), 753–789. 10.1210/er.2008-0014. 19837868

[R48] Teramo K, Hallman M, Raivio KO, 1980. Maternal glucocorticoid in unplanned premature labor. Controlled study on the effects of betamethasone phosphate on the phospholipids of the gastric aspirate and on the adrenal cortical function of the newborn infant (Apr). Pedia Res 14 (4 Pt 1), 326–329. 10.1203/00006450-198004000-00013. 7375188

[R49] Tronick EZ, 2003. Things still to be done on the still-face effect. Infancy 4 (4), 475–482. 10.1207/S15327078IN0404_02.

[R50] Waffarn F, Davis EP, 2012. Effects of antenatal corticosteroids on the hypothalamic-pituitary-adrenocortical axis of the fetus and newborn: experimental findings and clinical considerations (Dec). Am. J. Obstet. Gynecol 207 (6), 446–454. 10.1016/j.ajog.2012.06.012. 22840973 PMC3485443

[R51] Weiss SJ, Niemann S, 2015. Effects of antenatal corticosteroids on cortisol and heart rate reactivity of preterm infants (Oct)). Biol. Res Nurs 17 (5), 487–494. 10.1177/1099800414564860. 25608523

